# Design and Validation of a Holographic Particle Counter

**DOI:** 10.3390/s19224899

**Published:** 2019-11-09

**Authors:** Georg Brunnhofer, Alexander Bergmann, Andreas Klug, Martin Kraft

**Affiliations:** 1 Photonic Systems, CTR Carinthian Tech Research AG, 9524 Villach/St. Magdalen, Austria; Martin.Kraft@silicon-austria.com; 2 Institute of Electronic Sensor Systems, Graz University of Technology, 8010 Graz, Austria; Alexander.Bergmann@tugraz.at; 3 Nanophysics & Sensor Technologies, AVL List GmbH, 8020 Graz, Austria; Andreas.Klug@avl.com

**Keywords:** Condensation Particle Counter, in-line holography, Hough Transform, coincidence correction

## Abstract

An in-line holographic particle counter concept is presented and validated where multiple micrometer sized particles are detected in a three dimensional sampling volume, all at once. The proposed Particle Imaging Unit is capable of detecting holograms of particles which sizes are in the lower μm- range. The detection and counting principle is based on common image processing techniques using a customized Hough Transform with a result directly relating to the particle number concentration in the recorded sampling volume. The proposed counting unit is mounted ontop of a Condensation Nucleus Magnifier for comparison with a commercial TSI-3775 Condensation Particle Counter (CPC). The concept does not only allow for a precise in-situ determination of low particle number concentrations but also enables easy upscaling to higher particle densities (e.g., $>30.000\;\frac{\#}{ccm}$) through its linear expandability and option of cascading. The impact of coincidence at higher particle densities is shown and two coincidence correction approaches are presented where, at last, its analogy to the coincidence correction methods used in state-of-the-art CPCs is identified.

## 1. Introduction

A major topic in aerosol measurement is the detection of aerosol Particle Number (PN). Where Aerosol Electrometers are gaining more and more relevance for measuring high particle number concentrations, Optical Particle Counters (OPCs) still represent the most universal instruments to cope with low particle numbers. Single-particle detectors are commonly based on light scattering. Light scattering provides a direct measure of PN, particle size distribution and particle shape [1,2,3,4]. However, it also requires the magnification of nanoparticles to make them optically detectable. As standard optical microscopy is most often not eligible for commercial reasons, particles are counted after they are grown to micron size using standard instruments, such as Condensation Particle Counters (CPCs). Particle growth is achieved by passing the aerosol through a saturated vapor of a working fluid, followed by cooling in a condenser. This magnification stage is also referred to as Condensation Nucleus Magnifier (CNM). A subsequent counting unit is commonly based on nozzle designs to separate the grown droplets. These are passing a focused light beam where only one particle/droplet is illuminated at a time. The consecutive scattered light sequence is detected by a photo detector as an electrical pulse and each pulse is considered a single counting event. This standard counting approach requires to maintain a constant flow rate through the nozzle while measuring over a certain time interval to derive particle number concentration. At very low number concentrations the accuracy is limited by statistical error [5]. A true single-particle detection is only achievable when all particles in a sample volume are screened at once. Experiments are reported back to 1935 [6], where a camera photographed the number of suspended droplets in a known volume. Later, [7] and [8] developed automated instruments to determine the number of activated droplets and the aerosol concentration. However, due to the lack of continuous flow capability, these instruments primarily served for calibrations of newer types of CPCs, in which the particles are detected by photoelectrical means.

Recently, [9] proposed a new concept to operate an imaging system in an in-line holographic setup. Holography is not only capable of 3-dimensional (3D) imaging but also of resolving micro-sized objects without additional magnification optics and is thus more beneficial than photography. It also enables the possibility to monitor higher particle densities ($>30.000\frac{\#}{ccm}$) since the measurement volume can be arbitrarily expanded or cascaded by multiple systems at a linear scale. Although the holographic approach is already established in Holographic Particle Image Velocimetry (HPIV) [10] and is used to investigate aerosol particle morphology [11,12], to the knowledge of the authors it is the first implementation as a counting method, in particular for CPCs.

The herein presented work addresses the methodology, design, advancements and validation results of the aforementioned in-line holographic counting unit. It has to be mentioned that the following Imaging Unit is applicable in various measurement fields dealing with object sizes that are in the lower μm- range.

## 2. Methodology

**Figure 1:**
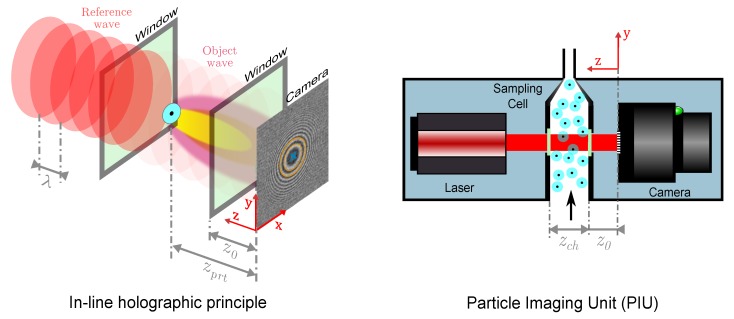
**Left**: in-line holographic principle where a single particle creates a fringe pattern at the camera plane. The detection is based on recognizing the set of concentric rings (in orange & blue); **Right**: schematic of the in-line holographic counting unit, subsequently called Particle Imaging Unit (PIU).

The holographic counting unit in Figure 1 is based on the in-line arrangement of [9] and is subsequently called Particle Imaging Unit (PIU). It comprises three core components: a laser, a sampling cell and an imaging camera.

Particles in the sampling cell of the PIU are illuminated by a reference plane wave, generated by the laser. A low coherence diode laser of the LNC-91CM-M60 series from Schäfter + Kirchhoff, with a wavelength λ of 635 nm and an adjustable output power $P_{max}$ of 1 mW, is used. Each particle in the sampling channel acts as a single point-like object which diffracts the incident plane wave to yield a spherical object wave. Both wave fronts propagate along the *z*- axis and interfere in a distance $z_{prt}$ at the detection- or hologram plane. The circular interference pattern formed at the hologram plane is also referred to as fringe pattern. The size of the fringe pattern is defined by the depth $z_{prt}$ along the illumination beam—[9,13].

Since every single particle yields a separate fringe pattern at the detection plane, the determination of particle count in the sampled volume is a matter of fringe pattern recognition. Of course, holographic imaging includes the full 3D information of the total sampling volume. However, that requires to reconstruct the whole volume and detect particles in a 3D space. Avoiding such typically iterative and computational intensive algorithms, the processing is done at the hologram plane where fringe patterns are detected and counted by simpler 2-dimensional (2D) image processing means.

A customized version of the Hough Transform (HT) of [14] is used which gives the total number of detected particles per acquired image (a paper on details is in preparation). It is a very well known feature extraction technique in digital image processing for detecting arbitrary geometrical shapes such as straight lines, circles or ellipses. The customized HT is developed to recognize fringe patterns as a set of concentric fringes.

## 3. System Realization

### 3.1. Sampling Cell

While the system in [9] was a proof of concept, where particles were sampled in open space, directly above the outlet of a particle source, the herein presented follow-up work also focuses on the design of a closed sampling cell to provide a well defined sampling volume of interest.

#### 3.1.1. Requirements

First, the Particle Imaging Unit is intended as an easy-to-replace alternative to counting units of state-of-the-art CPCs. As mentioned, these counting units directly follow the condensation section which are commonly prepared to attach a separation nozzle as its mandatory element. The presented sampling cell, thus, has to match the outlet geometry of the CNM (see in later) to be attached. Second, the temperature of such counting units are typically held slightly higher than the temperature of the condensation section to prevent the working fluid from condensing on the optical components. This is also crucial for the PIU and its cell windowing. An even more important reason implies a missing nozzle: in nozzle-based units the expansion of droplets is, to some extend, reinforced by adiabatic cooling through the pressure drop downstream the nozzle. Although the contribution to the final droplet size is negligible, the entailing acceleration of droplets prevents from evaporation before their eventual detection. In the proposed nozzle-free imaging approach, droplets are order of magnitudes slower and exhibit a longer travel path at which their size should retain. Conditioning the cell is therefore essential to maintain the supersaturated atmosphere and, thus, sustain the droplet size. And third, the optical axis between laser and camera must be insusceptible to mechanical vibrations and displacements. Dislocations in the range of the camera’s pixel pitch already have a big impact on the detectability of the imaging system. While static translations may be compensated through calibration, non-deterministic displacements are intolerable.

#### 3.1.2. Sampling Channel

**Figure 2:**
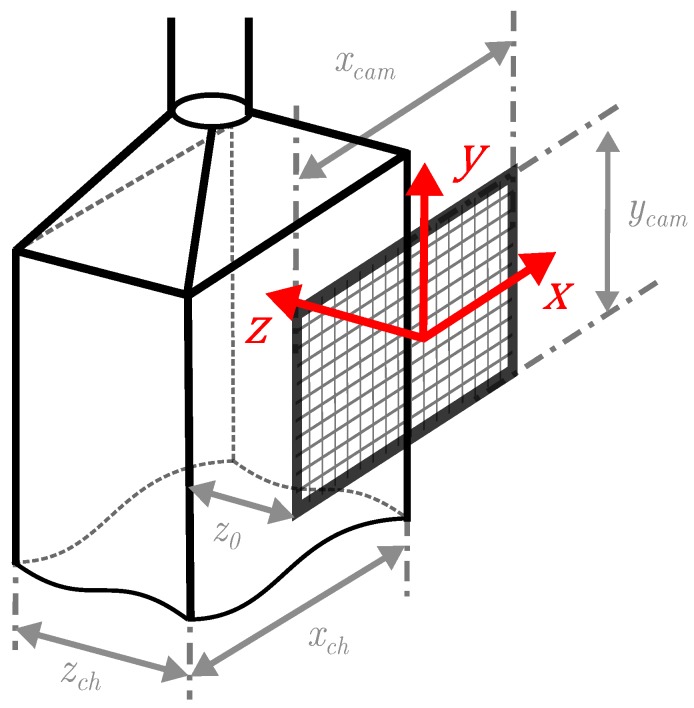
Sampling channel geometry of the PIU.

Figure 2 shows the sampling geometry of the PIU. It was dimensioned with the support of the Aerosol Particle Model (APM) presented by [13] and is subject to the following aspects: (i) a rectangular cross section is favourable due to its coplanarity; (ii) the width $x_{ch}$ of the sampling channel specifies the dimension of the imager; (iii) the depth $z_{ch}$ of the sampling channel determines the size of fringe patterns at the detection plane; and (iv) the resulting cross section affects the speed of droplets through the channel.

In detail:

(i) a volume imaged by a camera is formed by $x_{cam}\times y_{cam}\times z$, with *z* as the camera’s Depth of Field (DoF). Note that an Angle of View (AoV) does not arise as no lens is used and the sensor is illuminated with a collimated beam. The imaged volume is the basis for the later determination of particle number concentrations. Therefore, a rectangular cross section $x_{ch}\times z_{ch}$ of the sampling channel is meaningful to have a well defined sampling volume. This fact also reveals the advantage over nozzle-based counting systems as the sampled aerosol volume is known inherently.
(ii) the width $x_{ch}$ of the sampling channel should preferably match the width $x_{cam}$ of the camera to cover the whole sample flow. Since no camera objective is used in the current design to extend the Field of View (FoV), imager size and channel size are mutually dependent. Nevertheless, smaller camera outlines are possible because of the described volume definition, which is meaningful for later discussed reasons as well.
(iii) the depth $z_{ch}$ of the sampling channel also determines the DoF in terms of the holographic principle. The *z*-axis defines the size of particle’s fringe patterns on the detection plane. A strong variation in distances leads to a wide range of fringe pattern expansions. Thus, finding a meaningful range of distances which yield fringe pattern sizes with sufficient pixel resolution to be determinable and not occupying too much of the detection plane is essential for high density particle detection. A larger DoF additionally implies a higher coincidence probability of particles. This is why a small *z*- extend is favoured.
(iv) The cross section of the sampling channel affects the particle velocity which is of great importance for high quality imaging. As with faster traveling particles, the associated motion blur (discussed in Section 3.2) degrades the contrast and sharpness of imaged fringe patterns. As a consequence, the aerosol flow through the sampling cell needs to be reduced.

In conclusion, the dimensioning of the sampling channel is a compromise between the optimal aerosol flow rate, cameras (size, pixel pitch, exposure time) or imaging optics, and a meaningful distribution of fringe pattern expansions on the detection plane to achieve high detectable particle densities.

#### 3.1.3. Cell Windowing

As mentioned, the imaging unit is realized in an in-line holographic arrangement without imaging lenses. In order to facilitate a closed sampling cell, insertion frames equipped with proper windowing are inserted on both sides of the illumination path. N-BK7 broadband windows with a diameter of $1/2^{\prime \prime }$ are used in the given setup and, thus, determine the illumination cross section in the sampling channel. The general idea of insertion slots is to enable the possibility of easy exchange of different optical elements such as windows or lenses.

#### 3.1.4. Fabrication

To satisfy the requirements listed in Section 3.1.1, the sampling cell is a 3D- printed part made of AlSi10Mg, which is one of the standard alloys for Selective Laser Melting (SLM). It combines high specific strength with good thermal conductivity at a low coefficient of thermal expansion [15]. The latter is especially important as temperature fluctuations would lead to a noticeable misalignment of the optical path. In terms of thermal expansion, the sampling cell and the window insertion frames are matching components.

Where high thermal conductivity is of less significance or even unwanted, assembly parts are manufactured with an in-house Onyx 3D-printer.

### 3.2. Camera

As mentioned, the choice of an appropriate camera is subject to various criteria and in strong interrelationship to the geometry of the sampling channel, whether imaging optics is used, and ultimately the illumination of the light source.

#### 3.2.1. Requirements

The selection was also based on the APM and addresses the following requirements: (i) the imager, as exposed to bright light conditions, should provide a high dynamic range to still resolve the intensity distribution of particle’s interference patterns; (ii) the pixel pitch should be as small as possible to resolve single particles, ideally with no need for additional optical means; (iii) the imager size should preferably cover the volume of the entire sampling channel— Figure 1, right; and (iv) the exposure time should allow meaningful flow rates through the sampling cell to reliably operate the testing CNM as well as keeping motion blur within reasonable limits for good quality imaging.

#### 3.2.2. Technology Selection

As in in-line holography the camera is illuminated by the primary beam, high light intensity capabilities are required. A High Dynamic Range (HDR) is required to visualize high-contrast and extremely bright objects, but also darker image areas. Modern Complementary Metal-Oxide-Semiconductor (CMOS) sensors with a Global Shutter are available in different resolutions, are capable of Region of Interest (RoI) read out and allow high frame rates [16]. Considering these advantages and taken into account the emerging trend of manufacturers of focusing on CMOS sensor technology, it was preferred over Charge Coupled Devices (CCDs).

#### 3.2.3. Dimensioning

(i) high dynamic range capabilities are primary a question of the sensor technology as discussed in Section 3.2.1
(ii) the pixel size should be in the range of the expected particle/droplet size, preferably smaller to resolve it with several pixels. Unfortunately, the pixel pitch in CMOS sensors is in strong relationship to the carried out shutter principle due to packaging and complexity reasons of the sensor. That determining factor has to be taken into account as for moving object images, the shutter method has a significant impact. The two main principles are a Rolling- and a Global Shutter. Rolling Shutters require less internal logic and thus allow higher pixel densities. However, the drawback with less logic effort is that the exposure of the sensor is row-wise shifted, which adds additional shear to the imaged object—known as rolling-shutter-effect [16]. Preventing that effect, a Global Shutter facilitates simultaneous exposure of all pixels, but at the expense of pixel density.
(iii) the imager size, respectively the width $x_{cam}$ of the sensor chip is, to a major extend, predetermined by the sampling cell geometry. However, it is chosen slightly smaller than the extent of the sampling channel $x_{ch}$. This is reasonable from the fact that every beam shaping component (such as the sampling channel) causes diffraction patterns at beam forming edges. With a slightly larger sampling channel these unwanted patterns are moved out of the imagers field of vision. The camera’s capability of defining its RoI additionally allows for masking these patterns.
(iv) the exposure time directly affects the quality of holograms since overexposure of moving objects implies motion blur to the images. Quite to the contrary though, the fact of low intensity object waves necessitate long enough exposure times to obtain sufficient hologram contrasts. When aiming flow rates of Q=1 L/min and tolerating particles to pass only a few pixels (10–12 pixels were empirically determined to be tolerable and still allow high-contrast holograms), the required minimum exposure time is in the range of 10μs.

#### 3.2.4. Camera Selection

A UI-3082SE-M board-level camera from IDS was found to best meet the requirements listed before. It is based on a monochromatic CMOS sensor with a resolution of 2456×2054 pxl and a pixel size of 3.45μm. This gives a photosensitive area of 8.473 mm × 7.086 mm. It also provides the best compromise between small pixel size and Global Shutter availability. With the given camera dimenions and a sampling channel depth of $z_{ch}=4$ mm, fringe pattern are of sizes with sufficient pixel resolution to be determinable and do not occupy too much of the detection plane. This is a crucial prerequisite to distinguish patterns, especially at higher particle number concentrations.

As a trade-off and due to the camera’s limitation of supporting a minimum exposure time of $t_{exp}=25\;\mu$s, the flow rate through the sampling cell is reduced to $Q_{smpl}=0.3\;$ L/min. For all experiments in this work, the camera is operated at its minimum exposure time.

#### 3.2.5. Mounting

**Figure 3:**
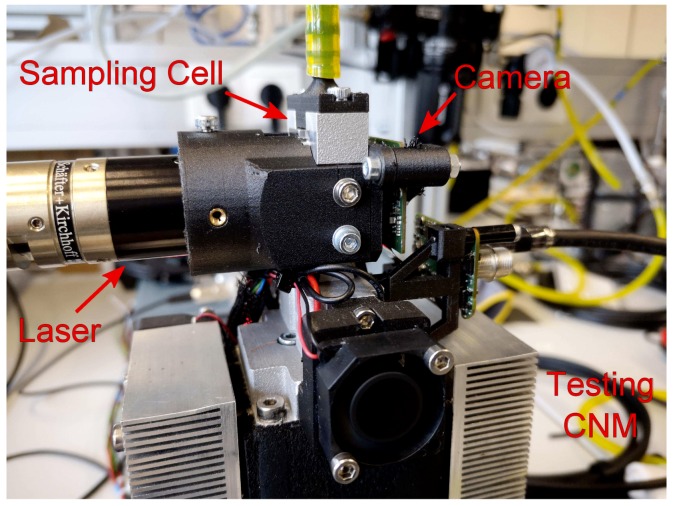
Particle Imaging Unit (PIU) ontop of the testing CNM outlet.

The sampling cell is designed to either attach customized camera mountings or 30 mm cage systems from THORLABS (the laser attachment is arranged in the same manner). Figure 3 shows the PIU mounted ontop of the testing CNM outlet. As can be seen, a boardlevel camera is used with the sensor chip and the periphery separated to two printed circuit boards and connected via ribbon cable. In that way, sensitive measurements are assured by decoupling vibrations and relief strain from the sensor chip.

The distance $z_{0}$ between the sensor chip and the inner sampling channel wall defines the minimum fringe pattern size and should be:

(1) $$z_{0}\geq \frac{b_{Pxl}\cdot D_{cam}}{\lambda }$$

to avoid aliasing effects [17], where $b_{Pxl}$ is the pixel pitch, λ the wavelength of illumination and $D_{cam}$ the shortest extend of the imager (here: $D_{cam}=y_{cam}$).

### 3.3. Image Processing & Particle Detection

#### 3.3.1. Image Preparation

As the intensity of diffracted waves are magnitudes lower than the direct beam, fringe patterns have a fairly low contrast and are hardly recognizable in the raw image. Furthermore, various system artifacts usually accumulate in the raw image. In order to suppress system artifacts, background correction is necessary where each imaged frame is subtracted by a calibration image (Section 4.2). Subsequently, a measurement image is referred to as a frame. It represents the more common term in the field of imaging, not least because the data acquisition in the experiments is conducted at a certain frame rate.

#### 3.3.2. Particle Detection Using a Customized Hough Transform

The Hough Transform is a common technique in digital image processing to find instances of objects with a certain class of shapes [18]. Typical applications are the identification of single and non-connected geometrical shapes in images such as lines, circles or ellipses. The transform is very powerful in finding imperfect, fragmented or occluded objects and is very robust in the presence of noise. This is the most important advantage here, because in frames of higher particle densities particles start to coincide and lead to overlapping fringe patterns.

In order to recognize a whole fringe pattern as a regular object, a customized version was developed which identifies fringe patterns as a collection of concentric rings with a common center point (a paper on details is in preparation). The output of the customized transform is the count of detected fringe patterns which directly relates to the count of particles in the sampled frame and, consequently, in the total sample volume. The parameterization is basically a matter of experimentally finding the best sensitivity for the detection of fringes. A too low sensitivity may lead to missing counts, while a too high sensitivity confuses system artefacts with valid fringe patterns.

**Figure 4:**
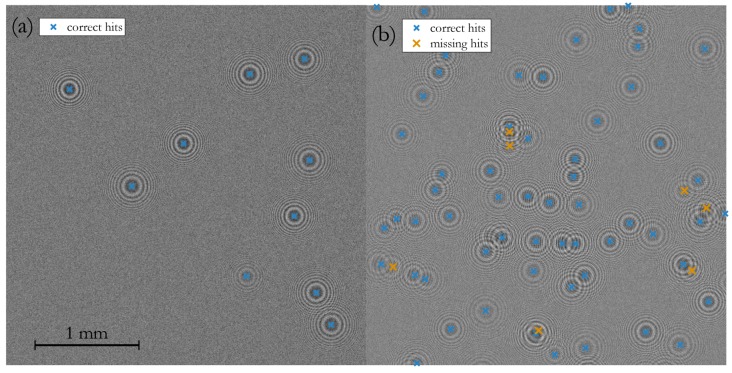
Zoomed segments of frames at: (**a**) low particle density of $78\;\frac{\#}{frame}$ and no missing hits; (**b**) medium particle density of $273\;\frac{\#}{frame}$ with missing hits. Because of intrinsic higher speckle noise in the right frame segment, the contrast is lower while the background noise level is increased.

The sensitivity of the customized HT is manually configured by visually inspecting two general scenarios in Figure 4: (a) in low particle density frames the algorithm is prone to misinterpreted irregular background noise as regular fringe patterns. Here, single detection hits are checked for correctness; (b) at very high particle densities, selected areas of strong fringe pattern overlaps are reviewed upon over- or underestimation of the counting rate. In such situations, multiple overlaps lead to wrong interpretations of the fringes and, hence, invalid hits.

A suitable configuration of the customized HT is a compromise between low and high particle density frames, especially as with rising particle load multiple scattering results in lower-contrast fringe patterns.

## 4. Experiment

### 4.1. Setup

**Figure 5:**
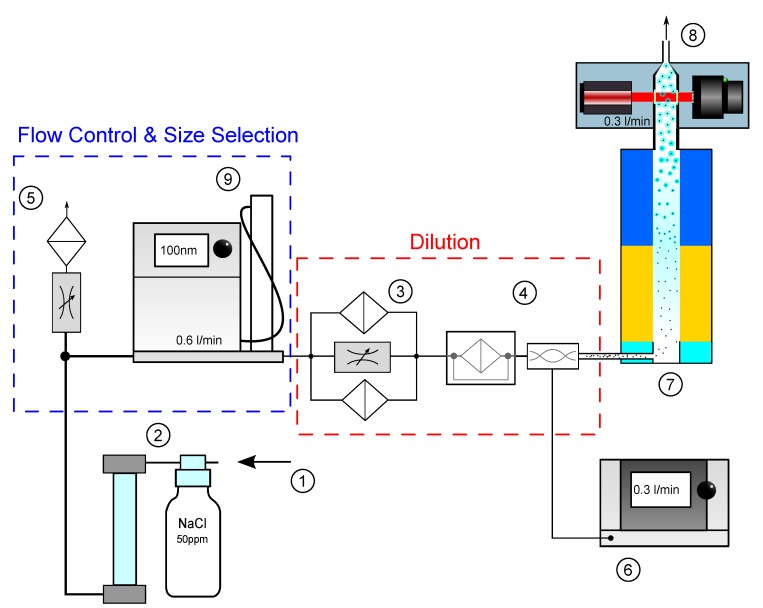
Experimental Setup for measurement of particle concentration.

Below, the main components of the experimental setup, as shown in Figure 5, are listed and described in detail in the following paragraph:

➀ Flow controlled pressurized air inlet
➁ Atomizer + Diffusion Dryer
➂ Dilution Bridge
➃ Bifurcated flow diluter + laminar mixer
➄ Bypass + mass flow fine tuning
➅ Referencing Condensation Particle Counter (CPC)
➆ Testing Condensation Nucleus Magnifier (CNM)
➇ Particle Imaging Unit (PIU) + Aerosol outlet

The testing CNM ➆, equipped with the Particle Imaging Unit ➇, was operated in pressurized mode where the aerosol is fed to the aerosol inlet through the following Flow Control & Size Selection, and Dilution setup: with an ATM220 Atomizer from TOPAS ➁ a test aerosol was atomized using a 50 ppm NaCl solution. The particle size distribution rated a geometric mean diameter of $d_{g}=53.6\;$ nm. The reference device ➅, a TSI-3775, has a 50% cutpoint at $d_{50}=4\;$ nm. The testing CNM, however, is a non-calibrated prototype with a cutpoint at around $d_{50}=30\;$ nm. A quantification of the counting results of the PIU is only meaningful if the reference CPC as well as the testing CNM have the same particle growth characteristics. To allow comparability and minimize particle size dependent detection efficiency, a TSI-3082 Electrostatic Classifier ➈ was used to select particles of size d=100 nm, for which both condensation units ensure highest counting efficiency.

Pressurized Air (PA) ➀ at 1.5 bar was supplied to the atomizer. The total flow rate through the dilution section was set to 0.6 L/min by venting overflow aerosol to the ambient over an additional needle valve ➄. In that manner, an equivalent portion of 0.3 L/min was drawn by the reference and the remaining portion was let through the testing CNM (and also allowing highest-contrast holograms as explained in Section 3.2.3). Dry air was assured by an oil trap connected directly after the laboratory gas supply; ➂ a controllable dilution bridge, consisting of multiple parallel High Efficiency Particulate Air (HEPA) filters and a needle valve to set the dilution, was used as an adjustable pre-dilution stage; ➃ a bifurcated flow diluter from [19] with a fixed dilution rate of 1:17 acted as secondary dilution stage to allow fine adjustment of particle number concentration over the entire dilution path. Note that the total filter area of the upstream dilution bridge was much higher and deliberately placed in front.

A Graphical User Interface (GUI) was developed to collect the measurement data of both counting units simultaneously. It triggers the imaging of the PIU ➇ and requests new samples of the TSI-3775 ➅ at the same time. New data acquisition was conducted manually.

### 4.2. Calibration of the PIU

**Figure 6:**
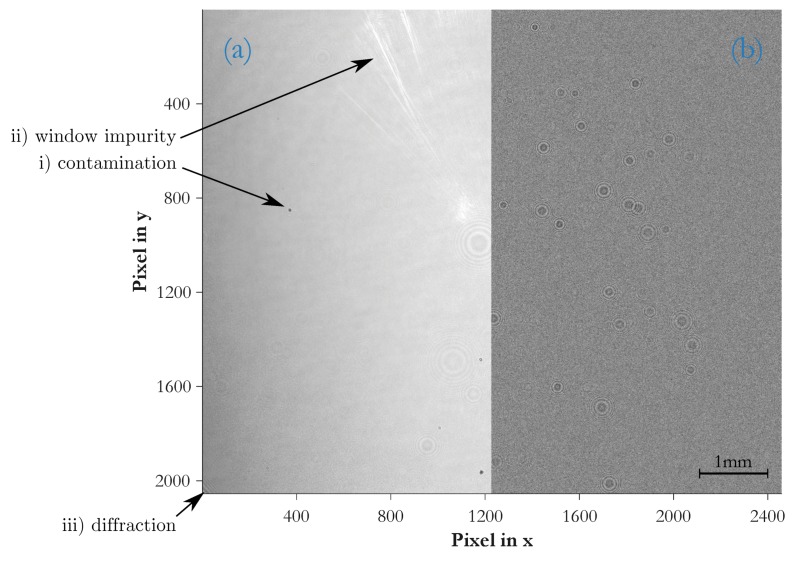
Imaged Hologram Plane: (**a**) left half of sampled volume as raw image. (**b**) right half of sampled volume as background corrected image

Calibration of the system implies to acquire the idle conditions of the imaging unit, when no particles are present in the optical path and, thus, subtracting system artifacts. Before each new measurement series, “zero-particle” images are acquired, where the bypass path of the dilution bridge ➂ is closed with the needle valve and the total aerosol flows through the HEPA filter section. The sampling flow through the testing CNM ➆ remains unchanged to calibrate under regular working conditions. Multiple calibration frames are averaged to a static background image which is subtracted from every measurement image—see the background correction of a particle measurement in Figure 6b.

As outlined in Section 3.1.1, calibration only compensates for static artifacts while non-deterministic effects are difficult to counteract. Long term drifts, as primarily originating from optical path misalignment, are compensated by dynamic calibration. In this approach, each new measurement frame adds a weighed contribution to the static background correction.

### 4.3. Variation of Particle Concentration

The detection- and counting efficiency of the PIU ➇ was valdidated with a sweep of particle number concentrations. For referencing, the TSI-3775 ➅ was sampling at the same flow rate of 0.3 L/min and in parallel to the testing CNM ➆. Before each measurement series, the particle size distribution of the atomized NaCl solution ➁ was set to a 100 nm mode with the Electrostatic Classifier ➈ to operate both condensation units with particle sizes where particle growth is guaranteed. After calibration of the PIU, the particle concentration was manually ramped by adjusting dilution with the needle valve of the dilution bridge ➂. The concentration was step-wise increased and monitored with the reference TSI-3775. The count rate of the PIU needs to be determined in a post-processing step since the customized HT is not optimized for real time applications yet. Hence, only a visual inspection was possible during measurements if the count rate in the sampling cell of the PIU apparently changed. After the concentration at the reference CPC ➅ stabilized, a new measurement sample was manually acquired with the aforementioned GUI.

## 5. Measurement Results & Discussion

### 5.1. Image Acquisition

Figure 6 depicts the imaged hologram plane of a measurement sample and should illustrate the difference between a raw image and the background-corrected result. The left side of the figure is the raw image and correlates to the left side of the sampled volume. The right side of the figure is the background-corrected result, ready for particle detection and correlates to the right side of the same sampled volume. Various system artifacts accumulate in the raw image: (i) contamination of optical elements in the illumination path; (ii) wear of the sampling cell windows; and (iii) diffraction patterns, caused by beam forming edges such as the window frames.

(i) the outer laser lens, the sampling cell windows and the sensor chip are under ambient conditions and, thus, prone to contamination such as particles, working fluid residues or residues from insufficient optics cleaning. This is visible where small and intense spots originate from particles sticking onto the sensor chip, and large and rather faint fringe patterns indicate impurities on either the laser lens or the cell windows.
(ii) the partial halo in the upper left image area comes from the sampling cell windows and may be caused by impurities, wear or adhesive residues.
(iii) the lower left corner reveals diffraction patterns from the window frame.

### 5.2. Determination of Particle Number Concentrations

**Figure 7:**
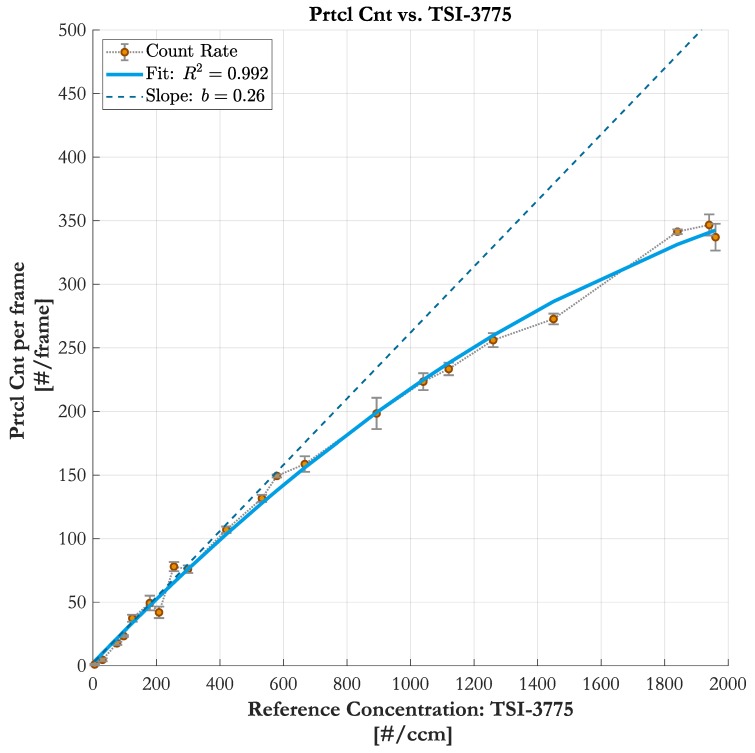
Comparison of the counting rate of the Particle Imaging Unit (PIU) to the monitored particle number concentration. The slobe *b* of the fit can be interpreted as the correlation factor.

The measurement result in Figure 7 shows the correlation between particle number concentration, monitored by the reference TSI-3775, and the particle count, detected by the PIU ontop of the testing CNM. Each data point is the averaged counting rate over multiple acquired frames at a camera frame rate of 23 fps. With 3 frames per measurement point the total sampling time per point is about ${\bar{t}}_{s}=130\;$ ms and therefore similar to the period settings of the moving average filter of the TSI-3775 [20].

#### 5.2.1. Correlation between the Particle Imaging Unit and the TSI-3775

A linear increase of particle number concentration means a linear increase of particle counts in the sampling channel. However, 2D imaging systems suffer from object’s shadow areas where there is no direct sight. The correlation curve in Figure 7 reveals the phenomenon of particles coinciding along the optical path at higher concentrations. It is fitted with a polynomial regression function of 2nd order, where the second coefficient is the slope of the regression. The slope b=0.26 can be interpreted as the correlation factor between the reference CPC and the imaging-based counting system.

The counting rate in the lower concentration range shows good linearity. In this region, less than 200 particles are present in the sampling volume and spatially well distributed to generate pronounced and well separated fringe patterns at the detector (see in Figure 4a). At higher particle densities, starting at around $600\;\frac{\#}{ccm}$, the probability of coinciding particles and, thus, overlapping fringe patterns rises (see in Figure 4b).

#### 5.2.2. Conversion to Concentration

The particle number concentration is determined by:

(2) $$C_{N}=\underset{\mathrm{flow}\text{-}\mathrm{based}}{\underset{︸}{\frac{N_{\tau }}{Q\cdot \tau_{s}}}}=\underset{\mathrm{volume}\text{-}\mathrm{based}}{\underset{︸}{\frac{N}{V_{s}}}}$$

where its formulation is either flow-based, with $N_{\tau }$ the particle count in a certain sample time interval $\tau_{s}$, or volume-based, with *N* the particle count in a given sample volume $V_{s}$ for visual systems. In contrast to flow driven CPCs, which require a constant flow rate *Q* as well as a known sample time interval $\tau_{s}$ to obtain particle number concentration $C_{N}$, an imaging system has the great advantage to inherently provide the concentration. The sample volume is a known quantity, defined by the FoV of the camera and the DoF, which is preset by the sampling channel depth $z_{ch}$ (see in Section 3.1.2). For the presented PIU, the volume rates roughly $V_{s}=0.24\;$ cm${}^{3}$.

From Equation (2) it is clear that the correlation factor *b* in Figure 7, between the flow-based particle number concentration result of the TSI-3775 and the volume-based counting rate of the PIU, is the volume $V_{s}$.

### 5.3. Limit of Detection of Particle Number Concentrations

The sensitivity of measuring particle number concentration is determined by the sampling volume and rates about $4.16\;\frac{\#}{ccm}$ for a single shot. When averaging multiple frames, the sensitivity may be easily increased by raising the camera’s frame rate. In this manner, an improvement of e.g.,: factor 20 is achieved with a frame rate of 20 fps (the integration interval is then 1s), which yields an improved sensitivity of $0.208\;\frac{\#}{ccm}$. Integration time, however, may also be increased while maintaining a constant frame rate and thereby improving sensitivity. It may not only be increased by averaging multiple frames but also by adapting the sampled volume. CMOS- imagers typically allow to configure a certain RoI which implies to alter the imaged sampling volume.

### 5.4. Detection Uncertainties

Because of the direct imaging of the total number of particles in a given volume the method is not only of first principle but also a true “zero- particle” monitor. However, several influences contribute to uncertainties in the unambiguous recognition of particle’s fringe patterns and consequently affect the unambiguity of particle number concentration.

#### 5.4.1. Minimum Particle Size and Particle Size Variations

The minimum resolvable size of particles is in strong relation to the FoV of the imager and the particle’s depth along the illumination path. The Rayleigh resolution limit [21] determines the smallest resolvable particle diameter as:

(3) $$d_{prt}>2.44\frac{\lambda \cdot z_{prt}}{x_{cam}}$$

with λ the wavelength of illumination, $z_{prt}$ the particle’s distance from the detection plane and $x_{cam}$ the width of the imager (Figure 2). In terms of the PIU it follows, that particles in the size range of 1–2 μm are theoretically resolvable. Therein, the shortest distance $z_{prt}=z_{0}$ yield the lower limit and $z_{prt}=z_{0}+z_{ch}$ the higher limit.

In practice, the minimum resolvable size of particles is also a matter of detecting sufficient intensity of the particle’s diffracted wave to outstand system noise [22]. Consequently, the dynamic range of the camera also contributes to the achievement of lower size limits which realistically lie in the range of 3–4 μm. Investigations may be a task for further studies.

#### 5.4.2. Illumination Inhomogeneity

The beam profile of lasers typically exhibit a gaussian intensity distribution. The utilized laser is designed to have a flat intensity profile over a certain cross section of the beam. Originally, an aperture defines that cross section and makes the laser suitable for applications using that specifc light beam shape. In the PIU the aperture needed to be removed to have the whole beam cross section available. As evident from the raw image in Figure 6a, the laser intensity at the edge of the channel is decreasing, resulting in a weaker particle illumation. As a consequence, a vanishing intensity of fringe patterns in that affected regions raises the probability of missing detection hits.

#### 5.4.3. Multiple Scattering

At high particle densities multiple scattering inevitably increases speckle noise, a granular interference of the scattered waves, which degrades the edge definition of fringes and limits the overall Signal-to-Noise Ratio (SNR)—compare Figure 4a with Figure 4b, where the intensity of the background level is slightly increased while the contrast is lower. The SNR was found in [23] to depend on particle diameter $d_{prt}$, the particle number concentration $C_{N}$ and the sample depth $z_{ch}$. The mean noise intensity $\langle I_{N}\rangle$ over *N* particles equals the standard deviation $\sigma_{N}$ of speckle noise and may be summarized to the simplified term:

(4) $$\sigma_{N}=\langle I_{N}\rangle =C_{N}\cdot \frac{\pi^{3}}{48}d_{prt}^{2}\cdot z_{ch}$$

It shows that for CPCs, where the particle diameter is uniform, the noise intensity linearly inreases with the particle number concentration.

#### 5.4.4. Coincidence of Particles

With rising particle densities in the sampling volume the probability of particles coinciding along the illumination path increases. With holographic alignments, of course, all objects are traceable. The typical approach is to apply backpropagation algorithms and detect objects in a reconstructed 3D volume. The herein favoured approach, however, is to omit such typically computational intensive algorithms and focus on the recognition of particles’ fringe patterns at the hologram plane. The tradeoff, as evident from Figure 4, is that fringe patterns start to overlap at higher particle densities as a consequence from the rising probability of coincidence. Standard 2D pattern recognition schemes fail to differentiate all valid hits and underestimated the actual counting rate.

The following section describes coincidence correction and its application in typical counting systems and proposes how it can be transferred to the given problem statement.

### 5.5. Coincidence Correction

In standard CPCs coincidence is a very well known limitation to reach high particle densities. The most common implementation to correct for coincidence is the so called Live Time Correction [24], where the total measurement time is deducted by the dead time, in which the system cannot accept any new count event. A count event is referred to as an electrical pulse, which a single photosensitive element—commonly a photodiode—is generating from each individual particle scattering occurrence. However, because of the small 3D viewing/measurement volume which is optically mapped to the photo detector, there is a finite probability of multiple particles coinciding in that particular volume. A statistical correction method based on a Poisson process was proposed by [25], which focuses on the probability of multiple events occurring in such per event dead times. The sampling channel of the herein described PIU can be considered as a large scale expansion of that aforementioned 3D measurement volume. Therefore, in analogy to a dead time in the electrical signal of standard counting units, a dead area may be introduced in the sense of images. Both approaches, Dead Area Correction as well as using a Lambert W function as proposed by [25] are potentially transferable.

#### 5.5.1. Dead Area Correction

Similar to the definition of dead time, where a system is blind to new count events in a certain period of time, an imaging system is blind to new events in a certain period of area, e.g.,: the scene behind an imaged object. The imaged object is, thus, considered as the dead area on an image in which the camera is blind for further count events. The image or frame itself is understood as the sample interval.

The common live-time corrected particle number concentration notation as in [24] may be adapted to:

(5) $$C_{a}=\frac{N_{m}}{z_{ch}\cdot \left(A_{cam}-f_{D}\cdot A_{d}\right)}$$

with $C_{a}$ the dead area corrected particle number concentration, $N_{m}$ the measured particle count per frame, $z_{ch}$ the depth of imaged volume, $A_{cam}$ the area which is spanned by the imager, $A_{d}$ the total dead area and $f_{D}$ a dead area correction factor. For every fringe pattern a certain per event dead area is defined in which an overlap of one or multiple patterns is not separable by simple image processing means. This area is a fraction at the center of the pattern and will be determined later in this section. As the expansion of fringe patterns is depending on the particle’s distance along the illumination path, a dead area correction factor $f_{D}$ takes into account the *z*- dependency.

#### 5.5.2. Poisson Process—Lambert W Function

The rate of particles flowing through the sampling channel occurs at random and, hence, follow a Poisson process. The Poisson distribution describes the probability of a given number of events occurring in a fixed interval of time, distance, area or volume. As particles are mapped to a detector plane, coincidence can be described as the probability of overlapping fringe patterns.

The probability *P* of *x* events occurring at a given rate $N_{a}$, in a certain area Ω is given by:

(6) $$P\left(X=x\right)=\frac{e^{-\mu }\mu^{x}}{x!}$$

(7) $$\mu =N_{a}\Omega$$

In analogy to [25], the probability that no event occurs in a certain fraction of the detector area, Ω, is:

(8) $$P\left(X=0\right)=e^{-\mu }=e^{-N_{a}\Omega }$$

and leads to the probability that one or multiple fringe patterns would be coincident at the actual count rate $N_{a}$:

(9) $$P\left(X\geq 1\right)=1-P\left(X=0\right)=1-e^{-N_{a}\Omega }$$

In order to express a relation between the measured count rate $N_{m}$ and the actual count rate $N_{a}$, Equation (9) may be solved to:

(10) $$1-e^{-N_{a}\Omega }=1-\frac{N_{m}}{N_{a}}\Rightarrow N_{m}=N_{a}e^{-N_{a}\Omega }$$

(11) $$\mathrm{with}\;\Omega =\frac{A_{d}}{\hat{A}}$$

where the occupied detector area Ω is the fraction of the total Dead Area $A_{d}$ over the total detector area $\hat{A}$. The per event dead area is considered as a certain centered part of fringe patterns in which one or multiple overlapping patterns are not separable by simple image processing means.

Since Equation (10) cannot be solved analytically, [26] proposed the so-called Lambert W function to correct for Dead Times in a counting process. The Lambert W function is defined as:

(12) $$y=W\left(x\right)\Leftrightarrow x=ye^{y}$$

and may be adapted to:

(13) $$N_{a}\cdot \Omega =-W\left(N_{m}\cdot \Omega \right)$$

where the left and right sides of Equation (10) are multiplied by −Ω.

#### 5.5.3. Definition of Dead Area & Practical Approximation

As the spatial extent of patterns originate from particles’ positions along the illumination path, a strong variation of pattern sizes occurs, while implicating an event-dependent dead area. The capability of separating events strongly depends on the applied image processing algorithms and has to be evaluated individually.

In the case at hand, the customized HT recognizes patterns by accumulating votes of circle center points and circle radii. When fringe patterns start to overlap, the accumulation points of votes in the Hough space also start to merge and, thus, span a certain dead area in the Hough space.

For sake of simplicity, particles in the sampling channel are assumed to be uniformly distributed. This allows to work with a mean fringe pattern size as if caused from a particle positioned halfway the sampling channel’s depth $z_{ch}$. As the recognition of patterns is based on circle extraction, the smallest detectable unit unaffected by overlaps should be the innermost circle. It is also the most pronounced one and yields the major contribution to the recognition.

In a first and simplified approach, the innermost circle may be approximated as the per event dead area as it represents the smallest resolvable fringe pattern indicator.

#### 5.5.4. Result of Conincidence Correction

The Aerosol Particle Model presented in [13] was used to determine the average fringe pattern size which is expectable from the geometries of the PIU. From the simulation, the innermost circle was found with an approximated diameter of $\delta_{d}=60\;$ pxl and is considered as the determining parameter, spanning a circular shaped per event dead area Ω.

**Figure 8:**
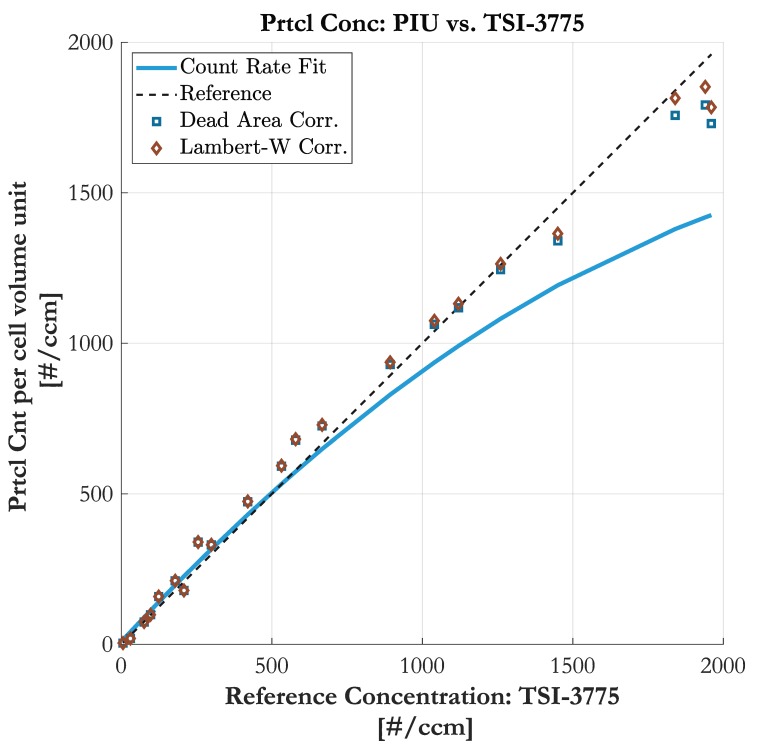
Comparison of particle number concentration obtained by the Particle Imaging Unit (PIU) and the TSI-3775 (uncorrected in blue). The concentration is coincidence-corrected by *Dead Area* and *Lambert-W* correction, both with a mean dead area diameter of $\delta_{d}=$ 60 pxl.

Figure 8 shows the trend of particle number concentration, once corrected by Dead Area Correction and once corrected with the Lambert-W function. Both approaches reveal good accordance to the actual reference trend of the TSI-3775. In the lower concentration region the correction effort is very low and the output of both methods equal. At higher count rates the Dead Area Correction tends to underestimate due to its simple nature of deducting “live volume”. In contrast, the Lambert-W function is based on statistical correction and takes into account the rising probability of multiple hidden particles in a consistent sampling volume. Nevertheless, both methods classify as suitable coincidence correction means. The results also confirm the transferability from time-domain-based counting units to imaging-based units.

## 6. Conclusions

A novel Holographic Particle Counter design is presented and validated. Introduced as Particle Imaging Unit, the counting unit is based on an in-line holographic arrangement and can be used to determine particle number concentrations of particle sizes that are in the lower μm- range. As the presented concept is a direct and volume-based imaging system, no flow rate measurement is required which is advantageous over standard light scattering methods of CPCs. Particles in the sampling volume are detected all at once. The particle number concentration is inherently determined, which makes it a simple and cheap standalone “single-particle” counting unit on the one side, but also a true “zero-particle” monitor. Instead of using backpropagation algorithms to detect all particles in the sampling volume, a typical 2D pattern recognition method is used to count particles at the hologram plane. At low particle densities up to $600\;\frac{\#}{ccm}$ the count response of the customized HT is linear and makes the counting approach perfectly suited for precise low concentration monitoring. At rising particle densities, the probability of particles coinciding in the sampling volume rises which lead to overlapping fringe patterns at the hologram plane. The resulting coincidence affected counting rate follows the same rules as time-based counting units of standard CPCs. Therefore, two coincidence correction methods are transferred to the 2-dimensional domain where its analogy to the time domain is shown and proven. Since the total sampling volume is scanned at once, an expansion of the volume may be realized to an arbitrary extend and thereby the range of measurable particle concentrations may be linearly upscaled. The sensitivity of the system in single shot mode is about $4.16\;\frac{\#}{ccm}$ and is easily increased by a factor of 20 and more by increasing the camera’s frame rate or the overall sampling period.
